# Decoding the Puzzle of Male Infertility: The Role of Infection, Inflammation, and Autoimmunity

**DOI:** 10.3390/diagnostics15050547

**Published:** 2025-02-24

**Authors:** Romualdo Sciorio, Lina De Paola, Tiziana Notari, Silvia Ganduscio, Patrizia Amato, Laura Crifasi, Daniela Marotto, Valentina Billone, Gaspare Cucinella, Antonio Perino, Luca Tramontano, Susanna Marinelli, Giuseppe Gullo

**Affiliations:** 1 Fertility Medicine and Gynecological Endocrinology Unit, Department Woman Mother Child, Lausanne University Hospital, 1011 Lausanne, Switzerland; sciorioromualdo@hotmail.com; 2 Department of Anatomical, Histological, Forensic and Orthopedic Sciences, Sapienza University of Rome, 00161 Rome, Italy; 3 Check-Up Poly-Diagnostic and Research Laboratory, Andrology Unit, 84131 Salerno, Italy; 4 Department of Obstetrics and Gynecology, IVF UNIT-AOOR Villa Sofia—Cervello, University of Palermo, 90127 Palermo, Italy; 5 Rheumatology Unit, ASL Salerno, 60th District, 84124 Salerno, Italy; 6 Rheumatology Unit, ASL Gallura, 07026 Olbia, Italy; 7 Département de Gynécologie-Obstétrique, Réseau Hospitalier Neuchâtelois, 2000 Neuchâtel, Switzerland; 8 School of Law, Polytechnic University of Marche, 60121 Ancona, Italy

**Keywords:** male infertility, infection, inflammation, epididymitis, oxidative stress, sperm quality, antisperm antibodies

## Abstract

**Background/Objectives**: Male infertility is a complex, multifactorial condition influenced by infectious, inflammatory, and autoimmune components. Immunological factors, though implicated in reproduction, remain poorly understood. This study aims to deepen the understanding of infections, inflammation, and autoimmune factors in male infertility, with a focus on immune-related disorders affecting the testes and epididymis—immunologically privileged but vulnerable sites. These factors can impair sperm quality through oxidative stress (ROS) and antisperm antibodies (ASA), further compromising fertility. **Methods**: A narrative review was conducted by analyzing scientific literature from the past 10 years conducted on PubMed using keywords such as “male infertility”, “autoimmunity”, and “inflammatory disease”. Studies focusing on testicular and epididymal disorders, immunological impacts, and therapeutic approaches were included. **Results**: Our research highlights that conditions like epididymitis, vasectomy, testicular trauma, and previous surgeries can trigger inflammatory responses, leading to ASA formation and oxidative stress. ASA, particularly sperm-immobilizing antibodies, inhibits sperm motility and migration in the female reproductive tract. Infections caused by sexually transmitted bacteria or urinary pathogens frequently induce epididymo-orchitis, a primary contributor to male infertility. While standardized methodologies for ASA testing remain elusive, assisted reproductive treatments such as intracytoplasmic sperm injection (ICSI), in vitro fertilization (IVF), and intrauterine insemination (IUI) show promise in overcoming immune-mediated infertility. **Conclusions**: This review underscores the critical role of infection, inflammation, and autoimmune responses in male infertility. It highlights the necessity of improving diagnostic methods, understanding immune-pathological mechanisms, and addressing medicolegal issues associated with male infertility. This knowledge could pave the way for innovative therapies, ultimately enhancing fertility outcomes, and mitigating the societal and legal repercussions of infertility.

## 1. Introduction

Infertility is involved in about 15% of couples worldwide [1], and male factors are implicated in about half of infertile treatments. The classical semen assessment can be extended with the detection of antisperm antibodies (ASA). Male immune infertility is defined as infertility caused by ASA [2]. Even though the most recent World Health Organization (WHO) manual for the examination of human semen reported that the only presence of sperm antibodies is insufficient for a diagnosis of sperm autoimmunity: it needs to establish that the antibodies interfere severely with sperm function and activity [2]. The sperm cells mature inside the seminiferous tubules of the testes. After the establishment of the immune system in early neonatal life, developing sperm must be protected from the immune system to prevent their recognition as foreign entities. Outside the seminiferous tubules, the testicular interstitium comprises a large number of immune cells, including mast cells, macrophages and dendritic cells; thus, it represents a unique immunosuppressed microenvironment by virtue of local immunoregulatory mechanisms [3,4,5]. Developing sperm are substantially enclosed in the interstitium and resident immune cells by the blood–testis barrier [4,5,6]. Sperm cells develop inside the blood–testis barrier, in a specialized milieu known as the adluminal compartment [5,6]. Tight junctions between the somatic Sertoli cells of the seminiferous tubules impede the free passage of proteins into the adluminal compartment and entry of immune cells and antibodies into the tubules. Immune privilege in the testis is a combination of the physical sequestration of developing sperm inside the blood–testis barrier (BTB), and local immunomodulatory factors that promote an immune-suppressed environment, as well as providing structural and nutritional support to the germ cells [3,4,5,6]. Therefore, alterations in such structures, as well as surgery, infection, and inflammation processes in the male reproductive tract are significant and might be associated and interfere with sperm function and the capacity to fertilize the oocytes [7]. The defined clinical entities include prostatitis, urethritis, seminal vesicles, orchitis, and epididymitis [8]. In addition, infections by sexually transmitted bacteria or caused by uropathogens are depicted as the most frequent cause of inflammatory conditions within the male genital tract [9]. Regarding the clinical impact of ASA, despite the extensive investigations recently conducted on the utility of the test, the management of immune infertility remains controversial. The sperm-agglutinating antibodies test was first announced in the serum and semen of humans in 1954 [10] and subsequently was associated with infertility by the 1970s [11]. The role and the impact of ASA alone on fertility outcomes has yet to be clarified. They can be identified in about 15% of infertile men and approximately 1–2% of fertile men [12]. Worldwide, ASA have been reported to be at comparable rates in different geographical areas and ethnically diverse infertile and fertile populations [13,14]. It is worth mentioning that autoimmune diseases might impair male reproductive health and fertility outcomes. This issue has been investigated in several papers, reporting that autoimmune diseases and immunosuppressive medications might induce change in hormonal levels and impair fertility outcomes [15,16]. Recent systematic reviews published have demonstrated a correlation between systemic autoimmune diseases in men and alteration in semen parameters, as well as DNA fragmentation, elevated gonadotropin levels, and varicocele [15,16,17]. Despite several authors having reported some evidence that autoimmune diseases are likely to impact male fertility, with variable effects on sperm quality, endocrine function, and reproductive outcomes, altogether the data currently available are scattered and heterogeneous, comprising small sample sizes, mixed treated and untreated populations, and restricted information about the treatment of these patients from a fertility perspective [16,17]. Furthermore, high-quality evidence on this topic is still lacking, and there are many treatments for autoimmune diseases and immunosuppressive medications for which no or very few studies exist with regard to their impact on reproductive function. There is an obvious need for deeper insight into testicular and epididymal immunopathologies and their contribution to male fertility [18]. Therefore, this review will focus on these issues, aiming to clarify immune infertility caused by ASA in men, as well as to explore the complex mechanisms underlying the pathogenesis of infection and inflammation in the male genital tract caused by elevated reactive oxygen species (ROS) [19], and ultimately to clarify the impact on couple reproductive outcomes.

## 2. Materials and Methods

A narrative review of the available scientific literature was conducted using the major databases PubMed and Scopus over the past 10 years. The keywords used included “male infertility” AND “inflammatory disease”, “male infertility” AND “autoimmunity”, “antisperm antibody” AND autoimmunity”. Inclusion criteria comprised only publications in English, original studies, and reviews relevant to male infertility associated with immunological factors, infections, or inflammatory diseases, as well as studies focusing on pathogenetic mechanisms at the testicular and epididymal levels. Studies examining the correlation between infertility and surgical interventions were also considered. Exclusion criteria applied to studies with unclear methodology, studies on non-human populations (except experimental animal models useful for understanding immunological mechanisms), and publications lacking abstracts or not accessible in full text. Articles demonstrating methodological rigor, including clinically or experimentally relevant data aligned with the study objectives, were prioritized.

Additionally, the review considered literature addressing the medicolegal implications of male infertility, with particular focus on cases related to autoimmune conditions or therapeutic and surgical complications.

The objective of this study was to analyze the current information on the correlation between inflammatory and autoimmune diseases and male infertility to identify knowledge gaps, research priorities, and potential therapeutic developments.

## 3. Results

### 3.1. The Structure and Immune Privilege of the Testis/Epididymis

The testes and epididymis are essential components of the male reproductive system, each with a unique immune environment necessary for defense against pathogens. This immune balance is essential to protect germ cells and sperm cells from autoimmune reactions on the one hand, while providing immunity against infection on the other. The testes are reproductive structures located within the scrotum, surrounded by a double-layered tunica vaginalis, and composed of multiple lobules, each containing seminiferous tubules supported by interstitial tissue. In the interstitial tissue, Leydig cells produce testosterone, along with macrophages and blood vessels. Spermatozoa are produced within the seminiferous tubules, which are lined with Sertoli cells. These cells assist in sperm maturation and provide nutritional support by regulating the transport of nutrients and trace elements across the blood–testis barrier (BTB). The epididymis connects the testis to the vas deferens and plays a crucial role in sperm maturation. It is divided into three regions—caput, corpus, and cauda—each with distinct immunological properties [20,21]. The testes and epididymis are unique in their ability to protect germs cells from immune system attacks. This sophisticated biological phenomenon, known as immune privilege, is essential for protecting male fertility as it prevents autoimmune reactions against sperm antigens that emerge during puberty [22]. Unlike most tissues, where immune cells can freely respond to antigens, the testis creates an environment that limits immune responses to avoid damage to developing germ cells. This balance between tolerance and defense reflects complex regulatory systems with significant implications for reproductive health and immunology. The testis achieves immune privilege through multiple complementary mechanisms. A critical component is the blood–testis barrier (BTB), a structure formed by specialized Sertoli cells that create tight junctions, physically separating germ cells from circulating immune elements, preventing immune cells and inflammatory molecules from crossing into the seminiferous environment. This immune-secluded environment is necessary because spermatocytes and spermatids emerge after immune tolerance to self-antigens is established; without the BTB, these cells could be recognized as non-self by the immune system. Additionally, Sertoli cells secrete immunosuppressive factors such as TGF-β and activins A and B, which inhibit T cell proliferation and help regulate the testicular immune microenvironment, that are essential for tissue homeostasis and male fertility. However, the BTB is more just than a physical barrier; it is highly dynamic, responding to hormonal cues and maintaining selective permeability to support spermatogenesis while limiting immune cell infiltration. The barrier’s adaptability is crucial for testicular homeostasis, emphasizing its ability to remodel under physiological stress [23].

In addition to the BTB, local immunosuppressive factors contribute to the unique testicular environment. Among these, transforming growth factor-beta (TGF-β) and interleukin-10 (IL-10) play pivotal roles. These cytokines modulate immune responses by inhibiting the activation of T cells and other pro-inflammatory pathways. TGF-β is indispensable in maintaining the anti-inflammatory milieu, ensuring that immune surveillance does not jeopardize germ cell integrity [24]. Moreover, Gualdoni et al. have focused on the overexpression of immunosuppressive enzymes such as indoleamine 2, 3-dioxygenase (IDO). IDO contributes to immune tolerance by degrading tryptophan, an essential amino acid for T cell proliferation, thus preventing potential autoimmune responses against germ cells. Another cornerstone of testicular immune privilege is the expression of Fas ligand (FasL) on the surface of Sertoli cells and germ cells. FasL binds to Fas receptors on T lymphocytes, triggering apoptosis and preventing the activation of autoreactive immune cells [25]. Regulatory T cells (Tregs) also play a fundamental role. These immune cells actively suppress autoreactive T cells and help maintain tolerance within the testis. Experimental depletion of Tregs leads to severe orchitis, demonstrating their necessity for immune balance. Tregs as central to preventing autoimmune responses that could otherwise compromise fertility [26].

The epididymis, a crucial component of the male reproductive system, facilitates sperm maturation, storage, and transport. Unlike the testis, which possesses a well-defined blood–testis barrier contributing to its immune privilege, the epididymis lacks such a barrier. Instead, it maintains immune privilege through a finely tuned balance between immune tolerance and defense mechanisms. This balance is essential to protect spermatozoa—recognized as foreign by the immune system due to their post-pubertal development—from autoimmune attacks, thereby preserving male fertility [26,27].

Epithelial cells in the epididymis secrete immunomodulatory factors, including β-defensins, that create a localized anti-inflammatory environment. Epididymis-specific proteins, such as clusterin, further enhance immune defense. This environment promotes tolerance towards sperm antigens while retaining the ability to mount immune responses against pathogens. Additionally, the epididymis exhibits regional specialization; the caput region fosters a more tolerogenic milieu, whereas the cauda region is more inclined towards pro-inflammatory responses [26,27,28].

The epididymis harbors resident macrophages (M1 and M2) and regulatory T cells that modulate immune responses. M1 macrophages are polarized by LPS alone or in combination with Th1 cytokines (e.g., IFN-γ, GM-CSF), producing pro-inflammatory cytokines such as IL-1β, IL-6, IL-12, IL-23, and TNF-α. M2 macrophages, polarized by Th2 cytokines (IL-4, IL-13), release anti-inflammatory cytokines like IL-10 and TGF-β3. In the initial segment (IS) and caput, macrophages are more abundant, whereas the corpus of the epididymis performs the dual function of establishing immune tolerance to descending sperm and defending against retrograde pathogens. In the cauda, phagocytosis, antigen processing, and presentation are more pronounced. Disruption of this immunological balance can lead to conditions such as epididymitis, resulting in infertility. Understanding the immune privilege of the epididymis is vital for developing therapeutic strategies aimed at treating male infertility and designing targeted immunosuppressive therapies that preserve the delicate equilibrium between immune tolerance and defense within this organ [27].

Research into the immunological privilege of the epididymis presents promising avenues for advancing fertility preservation and managing autoimmune diseases.

For instance, temporary modulation of the immune microenvironment in the epididymis could lead to reversible male contraceptive methods. By targeting immune checkpoints or cytokine pathways, spermatozoa could be transiently exposed to immune responses without permanent damage. Emerging evidence suggests that manipulating the PD-1/PD-L1 pathway, a key regulator of immune tolerance, may hold promise for such interventions [29].

Additionally, chronic epididymitis and sperm-targeted autoimmunity contribute significantly to male infertility. Therapies designed to restore immune privilege by enhancing local immunosuppressive signaling or bolstering regulatory T cell populations could mitigate inflammation and improve fertility outcomes. Advances in molecular diagnostics and localized immunotherapies are poised to transform clinical approaches to these conditions [27].

Finally, insights from epididymal immune privilege may extend beyond reproductive health. Strategies that mimic localized immune regulation could be applied to organ transplantation or the development of site-specific autoimmune treatments, where minimizing systemic immunosuppression is critical. These potential innovations make the epididymis a frontier for translational research, linking reproductive and systemic immunology [30].

Therefore, the immune privilege of the testis and epididymis reflects a delicate balance between immune tolerance and defense mechanisms. Continued exploration of these systems holds promise for advancing reproductive medicine and developing novel therapies for immune-related infertility and transplantation.

### 3.2. Non Infection, Infection, Inflammatory, and Autoimmune Diseases in Infertile Men

Over the past decade, numerous studies have been conducted on male infertility. These have raised a global concern: most men with fertility problems have suffered from inflammation in the genital tract [31].

There are several non-infectious pathologies of male reproductive organ that can negatively affect man’s reproductive potential (Table 1), and they can be divided into genetic, acquired and idiopathic causes [32,33]. They can be also divided into pre-testicular (diseases of the hypothalamus, pituitary gland, or other endocrine diseases), testicular (alterations in sperm production), and post-testicular (alterations of the seminal ducts and sexual dysfunctions).

Varicocele is considered the most common cause of male infertility. It is a condition that in fact occurs in approximately 40% of men with fertility problems. It has also been demonstrated that there is not a single triggering cause of varicocele, but there are mechanisms that contribute to each other such as oxidative stress, hypoxia, and consequently the lack of necessary nutrients. All these determine a chronic inflammatory state with production of cytokines that damage tissues and their functionality [32,34].

Non-infectious orchitis and epididymitis are characterized by inflammation of the testicle and the epididymis, respectively, but occur without a bacterial or viral infection. These conditions can lead to reproductive problems that can be permanent. Another problem that is often overlooked is that these conditions are in most cases difficult to diagnose due to their subtle clinical presentation and the presence of overlapping pathological conditions. Early diagnosis and appropriate treatment are the key to managing symptoms and preventing long-term damage. Over the years, it has been investigated how systemic autoimmune disorders, such as lupus erythematosus and various forms of systemic vasculitis, including Behçet’s disease, can negatively affect male fertility. It has been seen that these autoimmune diseases are able to involve the blood vessels of the testicle, epididymis, and excretory ducts, thus causing a deleterious local inflammatory disease. Among the non-infectious diseases that affect the male reproductive system, oncological diseases such as Germ Cell Tumors (most common type) should be mentioned. Testicular tumors are in fact able to determine an immune-mediated inflammatory response at the testicular level with associated extensive inflammatory infiltrates [37,38].

Another mechanism underlying male infertility is obesity. This can lead to infertility due to impaired testicular steroidogenesis [39]. In support of this, an association has been demonstrated between a diet rich in processed foods and poor in real nutrients and low sperm quality [40,41]. It is important to underline that the causes that determine infertility are numerous and are not limited to direct organic damage. Very often behind infertility are there psychological or socio-economic components that should not be underestimated, for they are a source of excessive stress for the patient that are ignored in most cases of infertility [41].

It is also true that infertility can be idiopathic or even unexplained. In these cases, there can be numerous physiopathological and biochemical processes underlying this disorder, such as oxidative stress. An observational study conducted at the University of Antioquia has shown how intracellular production of ROS and DNA fragmentation could be associated with idiopathic male infertility. In fact, a greater intracellular production of reactive oxygen species (ROS) and DNA fragmentation was detected in infertile patients compared to a group of fertile patients [42]. It has also been shown that male infertility may be related to exposure to toxic substances or drug use [43,44]. In addition to traditional known cytotoxic drugs, other drugs can have a negative impact on male fertility by different mechanisms, such as alteration of the hypothalamic–pituitary–gonadal axis or even through non-hormonal mechanisms. Some drugs can directly and indirectly cause damage to the male reproductive organ, causing sexual dysfunction and alteration in spermatogenesis [45,46]. Substances of abuse and psychedelics can also impair male fertility by causing damage to spermatogenesis, hormonal imbalances, or oxidative stress, with significant damage to other organs and even leading to death. Examples include anabolic steroids, alcohol, nicotine, cannabis, new psychoactive substances (NPS), volatile solvents, aerosolized chemicals. and fentanyl [47].

Despite ongoing research in this area, a significant proportion of cases of male infertility remain unexplained [48,49]. However, further research is needed to clarify the underlying pathogenesis, improve diagnostic accuracy, and optimize therapeutic strategies for these conditions.

### 3.3. Formation of ASA and Male Infertility

Spermatogenesis occurs inside the seminiferous tubules of the testicles, where the blood–testicular barrier, formed by the tight inter-cellular junctions between the Sertoli cells, prevents the spermatogenic cells from making contact with elements of the immune system and developing autoantibodies. Furthermore, the blood–testis barrier is permeable to small quantities of soluble sperm antigens capable of stimulating the production of T-suppressor lymphocytes, inducing a state of immunological tolerance, with a significantly reduced presence of T-helper lymphocytes in the seminiferous tubules’ interstitium. Alterations in the blood–testis barrier may allow the immune system to recognize spermatozoa as “non-self” and, consequently, to produce autoantibodies [5,50]. The seminiferous tubules converge at the rete testis, which acts as a transitional network connecting to the efferent ducts. From there, spermatozoa travel through the epididymis and the deferens before being mixed with prostatic and vesicular secretions in the urethra at the time of ejaculation. Studies involving intravenous injections of antisperm antibodies provide additional insights into immunological permeability. These experiments reveal that antibodies bind to spermatozoa emerging from the rete testis but not to those within the seminiferous tubules. This observation underscores a key difference: the rete testis exhibits greater permeability than seminiferous tubules, allowing both sperm antigen leakage and entry of soluble antisperm antibodies.

Despite this permeability, the concentration of spermatozoa within the rete testis is approximately half of that found in the distal male genital tract. This disparity suggests a natural mechanism of immunoprotection: the dilution of sperm antigens, combined with the limited vascularization of the rete testis, reduces immune exposure and minimizes the risk of immune responses targeting spermatozoa. Therefore, ASA formation in humans can occur at different levels: in the testicle, due to alteration of the blood–testis barrier, or during passage through the excretory tract. Main causes of ASA formation are: testicular or scrotal trauma (surgical, therapeutic, or iatrogenic), testicular torsion, testicular cancer, cryptorchidism, urogenital inflammatory conditions, obstruction, or varicocele [51,52] even if, in many cases, the origins of ASA are still unknown or idiopathic [53]. The effects of ASA on sperm functions are still controversial: Some studies report alterations in motility, capacitation, acrosome reactions, and fertilizing abilities of spermatozoon [54]. Whereas another study on many ASA positive patients demonstrated no correlation between motility and presence of IgA subtype of ASA [12]. Recent studies have not found a difference in embryo quality or fertility outcomes between couples with and without seminal ASA who are undergoing IVF with ICSI, suggesting there is likely to be little, if any, impact on embryo quality in this group [12]. Similarly, it is unclear whether ASA should be tested in all patients with asthenozoospermia or only in cases of asthenozoospermia with sperm agglutination and normal sperm concentration [13,55,56]. There is no clear evidence between sperm agglutination and ASA presence: other factors, beyond sperm antibodies, can also cause sperm agglutination [57], but more than one-third of patients with sperm agglutination had ASA, compared with less than 3% patients without sperm agglutination [58]. The 2010 WHO laboratory manual for the examination and processing of human semen describes sperm agglutinates as suggestive of the presence of ASA [2]. Briefly, the presence of ASA could be suspected in the presence of any suggestive history sperm agglutination regardless of whether they are associated with asthenozoospermia.

#### 3.3.1. Immunoglobulin Subtypes

ASA can be present in the blood serum and/or seminal plasma or attached to the surface of spermatozoa. The presence of circulating antibodies that do not interfere with sperm function has limited clinical significance. In contrast, antibodies adhering to the surface of spermatozoa, which alter their functionality and act as a physical barrier to sperm–oocyte interaction, have significant clinical relevance [59].

In seminal fluid, two immunoglobulin subtypes can be detected: IgA and IgG. IgA antibodies are of greater clinical importance compared to IgG antibodies, as more than 95% of cases with IgA sperm antibodies are also positive for IgG [2]. Immunoglobulin M (IgM) antibodies are rarely found [2], likely due to their larger size and their association primarily with the acute phase of infections.

#### 3.3.2. Testing for Antisperm Antibodies

There are sex differences in antisperm antibodies: In women, sperm are immunogenic as they are not inherently present in their own body, and ASA are strictly homologous antibodies. Women only develop circulating ASA [60]. In contrast, in men, ASA are produced as autoantibodies [61,62]. It critical for clinicians to recognize the sex difference in ASA when recommending tests for couples and when deciding on strategies if ASA are found to be positive in either partner. The World Health Organization (WHO) recommends performing ASA testing to detect sperm-bound antibodies during routine semen examinations [2]. Two tests have been widely used over the past 30 years to detect ASA on the sperm surface: the MAR (Mixed Antiglobulin Reaction) test and the IBT (Immuno Binding Test). The MAR test is a direct method performed on fresh and live spermatozoa incubated with latex beads coated with anti-human antibodies (IgA or IgG). If ASA are present, the anti-human antibodies on the beads bind to the antibodies on the sperm surface. As a result, motile spermatozoa appear coated with beads, which are observed under an optical microscope. The percentage of motile sperm with attached beads is then calculated [2].

This test provides information about the presence, type, and specific localization of antibodies on the sperm head, midpiece, tail, or all regions of the sperm. However, the direct test requires an adequate number of viable and motile spermatozoa. When these conditions are not met, indirect tests must be used. The IBT is an indirect method, applied when the number of motile spermatozoa is inadequate (less than 100) or in the absence of motile sperm. Sperm-free seminal plasma is incubated with ASA-free donor sperm, which are washed to remove original seminal fluid, and the MAR test for IgA or IgG is subsequently performed. The evaluation of MAR and IBT tests may vary depending on the operator’s subjective judgment. Recently, various methods for standardizing ASA evaluation have been proposed, including the application of computer-assisted sperm analysis (CASA) for MAR test analysis, enzyme-linked immunosorbent assay (ELISA) targeting anti-actin-like protein (ACTL)7 located in the acrosome and tail of mature sperm (recognized by antibodies in infertile serum) [63], and a novel protein biochip for screening serum ASA [64]. Improvements in ASA detection through standardized and user-friendly tests, supported by modern biotechnologies, offer promising tools for future clinical use

#### 3.3.3. Prevalence and Indirect Tests of Antisperm Antibodies

Antisperm antibodies (ASA) are immune proteins that mistakenly target sperm cells, causing damage and contributing to infertility. These antibodies may develop in response to infections, trauma, or other factors that compromise the integrity of the blood–testis barrier, allowing sperm to come into contact with the immune system. The prevalence of ASA in infertile men varies widely, ranging from 5% to 30% of cases depending on the population studied and the detection methods used [12]. The prevalence of ASA in infertile men is significantly higher than in the general population. In fertile men, antisperm antibodies are rarely detected, as the immune system typically does not generate a response against sperm cells due to mechanisms such as immune tolerance within the testes. However, in cases of infertility—particularly in men with conditions such as varicocele, epididymitis, or after vasectomy—the incidence of ASA is notably elevated [65].

ASA prevalence may also depend on factors such as the type of infertility (primary or secondary), the underlying cause, and the diagnostic methods used. For instance, men with autoimmune diseases or those with a history of trauma to the male reproductive tract are more likely to develop ASA. One study reported that 10–15% of infertile men tested positive for ASA, with higher rates observed in men with obstructive azoospermia or those who had undergone vasectomy [66,67].

Indirect tests are widely used to detect ASA in semen samples and provide crucial diagnostic information on male infertility. The most used techniques include the Mixed Antiglobulin Reaction (MAR) test, the Immunobead Test (IBT), and more recently, the Enzyme-Linked Immunosorbent Assay (ELISA).

- Mixed Antiglobulin Reaction (MAR) Test:

The MAR test is a direct method widely used for detecting ASA. It involves incubating sperm with antihuman globulin reagents that bind to any antibodies attached to the sperm. A positive result is indicated by visible clumping or agglutination of sperm, signaling the presence of ASA. This test is highly sensitive and specific, making it a preferred method for ASA detection [68,69]. The MAR test is particularly useful in clinical diagnostic settings, as it is a semi-quantitative test that can provide information on the percentage of spermatozoa bound to antibodies.

- Immunobead Test (IBT):

The IBT is another valuable method for identifying ASA. This test involves incubating sperm with small beads coated with antihuman globulin. If ASA are present, they bind to the beads, resulting in visible agglutination. The IBT offers a detailed assessment by identifying specific locations on the sperm where antibodies are attached, such as the head, midpiece, or tail. Due to its specificity and ability to distinguish various types of ASA, this test is being increasingly favored [70]. Although the IBT is a useful and precise test, in recent years it has become less available on the market, primarily due to its high cost and the need for specialized technologies.

- Enzyme-Linked Immunosorbent Assay (ELISA):

ELISA is an advanced technique capable of detecting and quantifying specific classes of antibodies, such as IgA, IgG, and IgM, in semen. While more commonly used in research, ELISA provides valuable information about the immune response to sperm, including systemic immune reactions that may not be apparent in traditional semen tests [13]. Although it is often considered a screening test rather than a direct clinical diagnostic test, it is primarily used in research settings rather than in routine clinical practice, as it requires more advanced equipment and more complex sample management.

Indirect tests such as the MAR, IBT, and ELISA are essential tools for diagnosis, providing increasing levels of specificity and sensitivity. Accurate detection of ASA facilitates better-informed decisions regarding treatment options, including assisted reproductive technologies such as intrauterine insemination (IUI) and in vitro fertilization (IVF) [71]. Understanding the role of ASA in male infertility is crucial for the development of effective therapeutic strategies. According to the latest WHO semen laboratory manual, the most used and recommended indirect tests for detecting ASA are primarily the MAR and, alternatively, the IBT [2].

#### 3.3.4. Effect of Antisperm Antibodies on Semen Parameters and Mechanisms of Infertility

The presence of ASA can alter various semen parameters and impair sperm function, ultimately impacting fertility. Recent studies have well-documented the role of ASA in infertility and highlighted their impact on semen quality and fertility mechanisms, which remains an active area of research.

Antisperm antibodies have been shown to negatively affect sperm count, motility, and morphology, contributing significantly to infertility. ASA can drastically reduce sperm motility by causing agglutination (clumping) or immobilization of sperm cells. Reduced motility impairs the sperm’s ability to navigate the female reproductive tract, thereby diminishing the chances of successful fertilization [66,72,73].

Sperm agglutination, where sperm clump together, further reduces the effective sperm concentration in the ejaculate. This clumping impairs sperm motility, making it increasingly difficult for the sperm to reach and fertilize the egg [66,67]. Moreover, the presence of ASA is linked to abnormal sperm morphology, with an increase in the number of morphologically defective sperm. Such abnormalities interfere with the sperm’s ability to bind to and penetrate the zona pellucida, a necessary step for initiating the acrosome reaction and achieving successful fertilization. When ASA block this interaction, fertilization often fails [53,74].

While ASA do not directly reduce sperm production, they affect sperm viability by triggering premature apoptosis or sperm death. This immune reaction reduces the overall number of viable sperm capable of fertilizing an oocyte, thereby further lowering the chances of successful conception [68,69,73]. Additionally, ASA can induce an inflammatory response within the male reproductive tract, creating a hostile environment for sperm survival and disrupting the reproductive microenvironment necessary for fertilization [69,74].

Interestingly, the formation of ASA has also been linked to the infection of certain viruses, including human papillomavirus (HPV) infection [75,76]. The presence of HPV on the sperm surface may act as a stimulus for ASA production, compounding the immune system’s impact on sperm functionality and fertility [72].

The close association between ASA and decreased fertility highlights the importance of ASA detection in diagnosing male infertility. Treatment options for managing ASA include corticosteroids, plasmapheresis, or assisted reproductive techniques (ART) such as intrauterine insemination (IUI) and in vitro fertilization (IVF) [52]. Studies on men undergoing intracytoplasmic sperm injection (ICSI) have shown no significant differences in fertilization rates, grade A embryo development, or pregnancy outcomes between patients with positive or negative ASA in seminal fluid [54].

#### 3.3.5. Pathogenesis of ASA

Antisperm antibodies are immune proteins that target spermatozoa, potentially leading to infertility in both men and women. In men, self-tolerance to sperm surface antigens does not develop during immunological maturation because spermatogenesis begins at puberty, long after immune tolerance to self-antigens has been established. Furthermore, the locations where sperm production, maturation, and semen transport occur are immunoprivileged. However, when the blood–testis barrier (BTB) or other immunological barriers are compromised, ASA formation may occur [67]. In men, this often results from trauma, infection, vasectomy, or surgical procedures that expose sperm antigens, which are typically sequestered from the immune system. ASA production can also arise due to congenital reproductive tract anomalies, but many cases remain idiopathic. This antigenic exposure triggers an immune response, leading the body to produce antibodies against sperm [67]. Although the seminiferous tubules were previously considered strictly anatomic barriers isolating sperm from circulating immune cells, it is now increasingly evident that both anatomic and immunological mechanisms maintain self-tolerance in the testes [67]. Tight junctions between Sertoli cells and epididymal cells form the structural foundation of the BTB, isolating sites of spermatogenesis and sperm transport. Additionally, low-permeability capillaries limit the migration of lymphocytes and antibodies into the seminiferous tubules where sperm is produced and matures.

Although not fully understood, these combined mechanisms ensure immunological tolerance of germ cells in healthy men. Disruption of these protective mechanisms underpins the formation of ASA, which can interfere with sperm functionality in several ways.

#### 3.3.6. Testicular Trauma or Surgery

Testicular injury is often considered a potential factor contributing to the development of ASA; however, the data present mixed findings. Several studies have shown that procedures such as testicular biopsy in cases of cryptorchidism, orchiectomy, or orchidopexy for testicular torsion, as well as surgical repair of testicular rupture, are not correlated with ASA formation [77,78,79]. Notably, this data comes primarily from pediatric populations, leading to the hypothesis that the absence of active spermatogenesis in these individuals may limit the exposure of sperm antigens necessary to trigger an antibody response.

In contrast, studies in adults have reported different outcomes. For instance, a trial conducted on adult males with testicular trauma, particularly in the context of varicocele, indicated a slightly increased risk of ASA development [51]. Furthermore, an observational study by Lotti and colleagues [80] examined the potential link between ASA and genital tract ultrasound abnormalities in men from infertile and fertile couples. Between 2012 and 2017, they analyzed 109 fertile men and 699 men undergoing infertility treatment. Their findings suggested a significant association between ASA and epididymal dysfunction, as detected via sonography, but not with testicular dysfunction. These results support the idea that chronic epididymal inflammation may play a greater role in ASA formation than testicular injury [80].

An intriguing area of investigation involves the potential for surgical trauma, particularly during surgical sperm retrieval, to contribute to ASA development by damaging the blood–testis barrier (BTB). Current evidence, however, does not support a significant association between procedures, such as testicular sperm extraction (TESE) or microdissection sperm retrieval, and the formation of new ASA in either males or their female partners. For example, a prospective trial by Ozturk and colleagues [81] examined couples after TESE and found no significant changes in testicular volumes, serum follicle-stimulating hormone (FSH), or testosterone levels before and after the procedure. Importantly, none of the patients or their partners developed significant ASA levels post-TESE. The study concluded that TESE does not induce ASA formation in either males or their female partners [33]. Other studies have similarly reported no incidences of new ASA development following surgical sperm retrieval [82,83,84].

#### 3.3.7. Varicocele

Varicocele is commonly associated with impaired fertility and abnormal semen parameters. The exact mechanisms through which varicocele affects fertility remain a subject of ongoing debate. Proposed mechanisms include scrotal hyperthermia, circulatory dysfunction, reactive oxygen species (ROS)-induced damage, and the formation of ASA [51]. A large observational study by Bozhedomov and colleagues conducted a multicenter, prospective study involving 1639 men from infertile couples and 90 fertile men. The study aimed to investigate the risks of immune infertility associated with varicocele and the impact of autoimmune responses on semen quality. The authors found that varicocele can act as a cofactor, increasing the risk of ASA formation. Notably, the likelihood of immune infertility following testicular trauma in varicocele patients was found to be two-fold higher. Additionally, varicocele was associated with a significant decline in semen quality, characterized by reduced sperm concentration, lower progressive motility, increased percentage of abnormal sperm forms, and higher levels of sperm DNA fragmentation [85]. Similarly, Bonyadi et al. reported that microsurgical varicocelectomy might lead to an increase in ASA, hypothesizing that the surgical procedure could impair fertility outcomes due to immune responses [36]. Furthermore, the presence of both varicocele and ASA may contribute to infertility in affected patients. Among men with varicocele, those who tested positive for ASA were more likely to have abnormal semen parameters, including reduced sperm number, motility, increased morphological abnormalities, elevated rates of premature acrosome reactions, and heightened sperm DNA fragmentation [85].

Microsurgical varicocelectomy has been shown to improve seminal parameters in both ASA-positive and ASA-negative patients. However, ASA-positive men generally have poorer semen analysis results compared to ASA-negative men, and they also tend to report lower natural pregnancy rates within the year following the procedure. Overall, these findings suggest that seminal ASA are predictive of poor sperm motility but do not necessarily predict improvements in semen quality after varicocele surgery [34,51,86,87].

#### 3.3.8. Inguinal Hernia and Repair

Inguinal hernia repair has been associated with the presence of ASA, primarily due to the disruption or damage to the blood–testis barrier (BTB), impaired testicular blood flow, injury or obstruction of the vas deferens, increased reactive oxygen species (ROS) production, and local inflammatory responses [87]. However, research on immunological infertility following inguinal hernia surgery remains limited. Generally, antisperm antibodies are known to adversely affect male fertility. A retrospective study by Negri and colleagues included 2258 infertile male patients who underwent urologic evaluation. Among them, 191 men had undergone inguinal hernia surgery. The authors aimed to investigate whether the presence of ASA, assessed via the mixed antiglobulin reaction (MAR) test, was more common in males who had undergone surgery compared to an unselected infertile population. The study found that men with a history of andrological surgery or groin herniorrhaphy had higher rates of seminal ASA [87]. The surgical technique used for hernia repair may also play an important role in the development of ASA and potentially impact fertility outcomes. Open mesh hernioplasty has been associated with a greater increase in serum ASA compared to laparoscopic or preperitoneal repair techniques. However, all surgical approaches have been found to improve semen quality to some extent [88,89]. Overall, these findings suggest that while inguinal hernia or its repair should prompt evaluation for ASA in infertile men, the surgery itself is unlikely to induce a significant autoimmune response as measured by serum ASA. A further study by Krnic and colleagues indicated that the mesh repair method did not lead to any clinically significant changes in testicular blood flow or notable alterations in the immune response [88,89,90].

#### 3.3.9. Cryptorchidism

Cryptorchidism is a condition describing undescended testicles that are not shielded in the scrotum but are instead located in the warmer abdominal cavity, which can disrupt normal sperm development which requires a cooler environment. This abnormal development may lead to the immune system recognizing sperm as foreign bodies, triggering an immune response that generates antisperm antibodies [91]. Generally, infertility in patients with a history of cryptorchidism is also associated with infertility usually resulting from oligo-asthenospermia, dysfunction in semen quality, and azoospermia [91]. However, a definite correlation between cryptorchidism and ASA has not been clearly demonstrated [92]. Some evidence coming from studies in the 1990s reported an association between cryptorchidism and serum ASA when compared with healthy controls [93]. A trial by Urry and collaborators investigated the incidence of antisperm antibodies in infertility patients with a history of cryptorchidism compared to general infertility patients. Among the men with history of cryptorchidism, 66% tested ASA positive compared to 2.6% of the control group. Furthermore, sperm motility was significantly reduced in men with ASA compared to those testing negative for antisperm antibodies. Also, as abnormal alterations in sperm morphology is generally the primary cause of infertility in patients with a history of cryptorchidism, it appears that the presence of ASA might also have increased in these patients, which may contribute to reduced fertility outcomes [74]. However, whether the above results represent a true difference or a difference in detection techniques has remained unclear [94,95,96].

#### 3.3.10. Relationship Between the Inflammation Condition, Obesity and Sperm Physiology

As previously mentioned, several studies have reported a relationship between chronic inflammation and obesity in men [97,98]. Adipocytes produce several compounds, including interleukins (IL-1, IL-6, IL-18) and tumor necrosis factor-α (TNF-α), which are classified as pro-inflammatory cytokines. These cytokines act as mediators of the inflammatory process by attracting macrophages. It is well established, particularly in animal studies, that pro-inflammatory cytokines can induce alterations in glucose homeostasis and insulin resistance, conditions commonly associated with obesity. Furthermore, these pro-inflammatory cytokines, such as TNF-α and IL-6, have been shown to be elevated in the seminal plasma of mice and in testicular tissue [99], potentially impairing the hypothalamic–pituitary axis (HPA) and negatively affecting fertility [100].

Systemic inflammatory diseases have also been shown to reduce testosterone production. The pro-inflammatory cytokine TNF-α inhibits LH function directly, leading to a reduction in testosterone production and contributing to male infertility [101]. Consequently, increased levels of chronic inflammatory cytokines in the serum of obese men may be associated with a decrease in androgen production at various levels of the hypothalamic–pituitary–Leydig cell axis.

In the testis, pro-inflammatory cytokines can directly impair the seminiferous epithelium. Sertoli cells are responsive to various pro-inflammatory cytokines, most notably IL-1 and TNF-α. These cytokines influence the production of junctional proteins such as zonulin/zonula occludens-1 (ZO-1), claudins, and actin–myosin cytoskeletal proteins. This disruption may compromise the integrity of the junctions between adjacent Sertoli cells, altering the seminiferous epithelium and negatively affecting sperm production [6,102]. Reduced expression of junctional proteins in Sertoli cells has been observed in several diet-induced obese animal models [103].

Sperm passage through the epididymal tract is essential for acquiring lipids and proteins necessary for sperm maturation and motility [104]. Obesity and pro-inflammatory conditions may alter the function of the epididymal epithelium by modifying the environment within the epididymis, changing the content of epididymosomes, and increasing the influx of neutrophils and macrophages into the epididymal lumen. This results in higher cytokine expression and epithelial apoptosis, which can impair sperm maturation and reduce fertilization ability [104]. In summary, pro-inflammatory cytokines produced within the testis and epididymis can disrupt the critical processes regulating spermatogenesis and sperm maturation.

#### 3.3.11. Oxidative Stress and Sperm Quality

Several reports have recently demonstrated that one of the major testicular impairments might be the increased production of reactive oxygen species (ROS) and oxidative stress, causing changes in protein production and DNA damage [105,106,107,108,109]. Increased ROS production is a condition in which free radicals are produced and released in the cellular compartment. These compounds can be highly reactive and can produce additional toxic chemicals, damaging cells and tissues, as well as impairing gamete physiology and embryo development [107,110]. Elevated ROS levels in the testis have been correlated with dysfunction in site-specific hypermethylation through the upregulation of DNA methyltransferases (DNMTs) or the formation of new DNMT-including complexes [111]. It is important to note that sperm epigenetic alterations could be secondary to additional factors, such as sperm manipulation or other characteristics associated with the patients [111,112]. Furthermore, an increase in seminal ROS has been correlated with alterations in sperm membrane potential, as well as chromosomal abnormalities, micronuclei formation, and increased apoptosis and DNA fragmentation [31,110,111,113]. Sperm DNA damage induced by ROS negatively affects embryo development, resulting in an increased risk of miscarriage, depending on the extent of single- or double-strand breaks in the sperm DNA and the ability of the oocyte to repair some of them following fertilization [114]. Elevated ROS levels may also influence the seminiferous tubules, Leydig cells, and spermatozoa, inducing a reduction in testicular steroidogenesis and lowered testosterone levels [113,114,115]. A correlation has also been reported between oxidative stress, increased lipid peroxidation, and changes in the body’s antioxidant functions. The sperm plasmalemma is particularly sensitive to lipid peroxidation due to its high polyunsaturated fatty acid content. Therefore, the formation of additional toxic lipid peroxidation products may damage the sperm membrane and mitochondrial proteins of the electron transport chain, thereby decreasing sperm motility and fertilization potential [116]. Sperm motility, acrosome reaction, and binding to the oocyte are critical features for fertilization and embryo development. Thus, elevated ROS levels might affect and injure membrane fluidity and integrity [107,108,109,110], impairing mitochondrial function and activities. Mitochondria are essential organelles in providing energy for sperm motility, and any metabolic disruption in the electron transport chain can significantly increase mitochondrial ROS production, affecting sperm quality [116]. Excessive ROS production can alter the metabolic function and activities of seminal enzymes, such as superoxide dismutase (SOD), catalase (CAT), and glutathione peroxidase (GPx), which play a critical role in protecting spermatozoa from oxidative stress. Damage to the plasma membrane and sperm quality negatively interferes with sperm physiology and fertilization potential [106,109,117]. The production of ASA could be associated with a chronic inflammatory response induced by oxidative stress, further damaging the spermatozoa and impairing their functionality. This process could exacerbate the damage caused by ROS, contributing to a vicious cycle that negatively affects sperm quality and fertility.

#### 3.3.12. Infertility Treatments

Another important area of research regarding infertility, its causes, and its consequences is represented by assisted reproduction techniques [71,118]. Intrauterine insemination (IUI) can bypass the cervical mucus and is recognized as an effective method to overcome poor cervical mucus penetration and the movement of sperm in samples with ASA. Barbonetti and colleagues [13] reported a live birth rate of 36.8% using IUI in patients with 100% positive ASA in their semen, which was significantly higher compared to the natural live birth rate of 4.5%. Alternative methods suggested for overcoming ASA in semen include assisted reproduction technologies (ART), such as standard IVF insemination or intracytoplasmic sperm injection (ICSI). Several studies have shown that ASA has no influence on fertilization rates, embryo development, clinical pregnancy, implantation, or live birth rates when ICSI is used for insemination [52,54,119,120]. Lu and colleagues analyzed nearly 500 treatments (399 IVF and 155 ICSI cycles) to examine the differences in fertilization and pregnancy outcomes associated with ASA-positive and ASA-negative men. The results showed lower fertilization rates (41.7% vs. 54.8%), poorer embryo development (18.9% vs. 35.2%), and lower live birth rates (25.8% vs. 42.5%) in patients undergoing IVF with ASA-positive semen compared to ASA-negative semen. In addition, couples with ASA-positive male partners had lower live birth rates with IVF (25.8%) compared to those who underwent ICSI treatment (47.4%). However, the difference was not observed in the ICSI group, where both ASA-positive and ASA-negative groups had similar success rates [52]. These findings, however, were not confirmed by other studies, which did not find a significant relationship between direct ASA levels and reproductive outcomes following either IVF or ICSI insemination [121]. Further prospective trials correlating pregnancy outcomes after standard IVF and ICSI in ASA-positive patients have shown significantly higher pregnancy and live birth rates with ICSI compared to regular IVF insemination [83]. Given the association between ASA positivity and greater success with ICSI over IVF, detection of ASA should be considered as an indication for prioritizing ICSI as the first-line treatment option [52,122].

### 3.4. Legal Framework

The medicolegal implications in the field of male infertility related to infections, inflammations, and autoimmune disorders can be numerous and significant. These may vary depending on the potential responsibility of a healthcare professional and the severity of the consequences on male fertility [123,124]. A delayed or incorrect diagnosis, for instance, could lead to a delay in initiating treatments, further compromising fertility. Failure to recognize or treat infections like chlamydia or gonorrhea could cause permanent damage to reproductive organs, resulting in infertility. If a causal link between the failure or delay in diagnosis and infertility is established, the healthcare providers responsible could face legal issues and be required to compensate the patient for the serious harm caused. Proper primary prevention, along with a thorough evaluation using targeted infectious disease tests and following medical guidelines, should be implemented in case of any doubts, without resorting to expensive and unnecessary defensive medicine.

Furthermore, some pharmacological treatments, immunosuppressive or anti-inflammatory therapies, surgeries, or assisted reproduction interventions might prove inadequate [125] and compromise fertility. These could be considered medical errors, carrying professional liability and a potential claim for biological damage compensation. Many procedures are not free from complications and significant side effects concerning fertility [126]. Legal disputes can also arise in cases of medically assisted reproduction that have failed due to immune disorders or previous therapies. Therefore, it would be helpful to clearly inform the patient and establish efficient and effective communication between patients and assisted reproduction centers, which helps define the limits of responsibility [127,128].

It is therefore important to base every medical treatment on validated medical literature and always provide accurate information regarding the risks, benefits, and alternatives of treatments. At this point, it is crucial to remember that proper information is achieved only through informed consent, which must include all its inherent characteristics: it should be clear, informed, contemporaneous, personalized, and voluntary. Patients, in the field of infertility as in other medical/surgical contexts, must be able to make autonomous decisions and give their consent to the proposed procedures with full awareness of the potential risks, including possibly infertility [129]. Patients who are inadequately informed, or in cases of deficient consent, may initiate legal proceedings and challenge their consent if they were not properly informed of the risks associated with diagnostic or therapeutic procedures. Furthermore, it is not uncommon for healthcare professionals to be verbally or physically attacked in cases of apparent negligence [130]. It is important to remember that the time spent on providing information is always part of the care process and, therefore, essential before medical procedures. Ensuring that the patient understands all available options and potential consequences is a necessary and preparatory step.

Additionally, it must be emphasized that even innovative or experimental treatments, such as certain genetic or immunological therapies, or emerging technologies like AI in medicine [131,132,133], can cause harm to individuals, including damage to fertility, which may sometimes not be fully documented. To avoid lengthy legal disputes in the case of harm, it is crucial to implement rigorous research protocols and maintain accurate, clear documentation.

In conclusion, medicolegal disputes can be minimized through clinical practice based on solid and scientifically validated protocols, comprehensive informed consent, complete and transparent documentation, and proper monitoring of patients.

## 4. Discussion

In this narrative, we aimed to describe the association between ASA and reproductive impairment following assisted reproductive technology cycles. ASA are heterogeneous and may interfere negatively with fertility, particularly by inhibiting sperm migration through the female reproductive tract. However, ASA can also have inhibitory effects during sperm–egg interaction, fertilization, and further embryo development [59,74]. Currently, ART procedures such as intrauterine insemination (IUI), in vitro fertilization (IVF), and intracytoplasmic sperm injection (ICSI) are considered effective techniques for resolving infertility in couples with suspected immune infertility or ASA.

It is evident that ASA-induced infertility and seminal fluid-induced fertility enhancement involve several different mechanisms, some of which need to be clearly identified. Therefore, the further development of reproductive medicine is necessary. A better understanding of these factors will improve diagnostic and therapeutic strategies, opening up new therapeutic avenues in the field of fertility.

Future research should focus on identifying novel biomarkers and therapeutic options aimed at more effectively addressing these often-underestimated causes of male infertility. Limitations in diagnosis and adequate therapy highlight the urgent need for a greater understanding of human epididymal and testicular immunopathologies and their association with male infertility. A significant obstacle to increasing knowledge in this area is the limited access to appropriate tissue samples. Additionally, immunological conditions often manifest well before the patient seeks fertility evaluation. In this sense, experimental data from animal models may offer an alternative approach to overcome the restrictions of studying humans.

## 5. Conclusions

In conclusion, while advances in assisted reproductive technologies have provided valuable solutions for male infertility associated with ASA, further research is essential to unravel the complexities of ASA-induced infertility. Improved diagnostic tools and more targeted therapeutic approaches are needed to enhance the success of treatment and provide better outcomes for couples affected by this condition.

## Figures and Tables

**Table 1:** Conditions of male genital system and their negative influence on fertility.

CONDITION	DEFINITION	ETIOLOGY	INFERTILITY MECHANISM
VARICOCELE [32,34]	Enlargement of the veins in the scrotum, usually on the left side, affecting testicular function.	Idiopathic, often related to faulty valves in the testicular veins, obstructing blood flow.	Testicular hypoxia and presents of ASA, spermatogenesis and testosterone secretion abnormalities, and testicular cytokine production.
TESTICULAR TORSION [35]	Twisting of the spermatic cord, cutting off blood flow to the testis.	May be spontaneous or due to trauma.	Ischemia due to compromised blood flow results in testicular necrosis if untreated, leading to infertility.
EPIDIDYMTIS (NON INFECTIOUS) [36]	Inflammation of the epididymis	Trauma, autoimmune diseases, or idiopathic causes.	Blockage of sperm transport or chronic inflammation can lead to impaired fertility.
HYDROCELE (NON INFECTIOUS) [36,37]	Accumulation of fluid around the testis, causing scrotal swelling.	Trauma, or idiopathic causes.	Pressure on the testis may cause impaired sperm production over time, though usually asymptomatic.
TESTICULAR CANCER [38]	Malignant tumors arising from testicular germ cells or stromal tissue.	Genetic factors, cryptorchidism, family history, or history of testicular trauma.	Impaired sperm production due to testicular destruction by the tumor, may require orchiectomy.
KLINEFELTER SYNDROME	A genetic disorder where males have an extra X chromosome.	Small testes, gynecomastia, infertility, delayed speech or motor skills in childhood, reduced libido.	Genetic testing (karyotype), hormonal tests (low testosterone, high FSH and LH), semen analysis.
OBSTRUCTIVE AZOOSPERMIA	Complete absence of sperm in the semen due to blockages in the reproductive tract.	Absence of sperm in semen, normal libido, and normal hormone levels.	Physical obstruction in the epididymis or vas deferens prevents sperm ejaculation.
AUTOIMMUNE ORCHITIS [36]	Inflammation of the testis due to the immune system attacking testicular tissue.	In isolation or with systemic autoimmune diseases (SLE, rheumatoid arthritis, sarcoidosis).	Immune-mediated destruction of Leydig and Sertoli cells, leading to testicular atrophy and impaired spermatogenesis.
AUTOIMMUNE EPIDIDYMITIS [36]	Immune-mediated damage to the epididymis	In isolation or with systemic autoimmune diseases (Morbus Behcet, systemic lupus erythematosus, Schönlein-Henoch purpura and other vasculitic disorders)	Obstruction and damage to the epididymis result in impaired sperm maturation and transport.
TESTICULAR VASCULITIS	Inflammation of the blood vessels of the testis, part of systemic vasculitis.	Associated with autoimmune vasculitides such as polyarteritis nodosa or Wegener’s granulomatosis.	Inflammation of blood vessels impairs testicular blood flow, leading to ischemia and impaired sperm production.
OBESITY [39]	BMI > 30	In most cases in the context of metabolic syndrome	Disruption of testicular steroidogenesis

## Data Availability

The raw data supporting the conclusions of this article will be made available by the authors on request.
